# Pneumonia and Pulmonary Thromboembolism Classification Using Electronic Health Records

**DOI:** 10.3390/diagnostics12102536

**Published:** 2022-10-19

**Authors:** Sinhue Siordia-Millán, Sulema Torres-Ramos, Ricardo A. Salido-Ruiz, Daniel Hernández-Gordillo, Tracy Pérez-Gutiérrez, Israel Román-Godínez

**Affiliations:** 1 División de Tecnologías para la Integración Ciber-Humana, Centro Universitario de Ciencias Exactas e Ingenierías, Universidad de Guadalajara, Guadalajara 44430, Mexico; 2 Unidad Médica De Alta Especialidad, Hospital de Especialidades, Centro Médico Nacional De Occidente, Guadalajara 44349, Mexico

**Keywords:** automatic clinical diagnosis, pneumonia, pulmonary thromboembolism, machine learning, BiLSTM

## Abstract

Pneumonia and pulmonary thromboembolism (PTE) are both respiratory diseases; their diagnosis is difficult due to their similarity in symptoms, medical subjectivity, and the large amount of information from different sources necessary for a correct diagnosis. Analysis of such clinical data using computational tools could help medical staff reduce time, increase diagnostic certainty, and improve patient care during hospitalization. In addition, no studies have been found that analyze all clinical information on the Mexican population in the Spanish language. Therefore, this work performs automatic diagnosis of pneumonia and pulmonary thromboembolism using machine-learning tools along with clinical laboratory information (structured data) and clinical text (unstructured data) obtained from electronic health records. A cohort of 173 clinical records was obtained from the Mexican Social Security Institute. The data were preprocessed, transformed, and adjusted to be analyzed using several machine-learning algorithms. For structured data, naïve Bayes, support vector machine, decision trees, AdaBoost, random forest, and multilayer perceptron were used; for unstructured data, a BiLSTM was used. K-fold cross-validation and leave-one-out were used for evaluation of structured data, and hold-out was used for unstructured data; additionally, 1-vs.-1 and 1-vs.-rest approaches were used. Structured data results show that the highest AUC-ROC was achieved by the naïve Bayes algorithm classifying PTE vs. pneumonia (87.0%), PTE vs. control (75.1%), and pneumonia vs. control (85.2%) with the 1-vs.-1 approach; for the 1-vs.-rest approach, the best performance was reported in pneumonia vs. rest (86.3%) and PTE vs. rest (79.7%) using naïve Bayes, and control vs. diseases (79.8%) using decision trees. Regarding unstructured data, the results do not present a good AUC-ROC; however, the best F1-score were scored for control vs. disease (72.7%) in the 1-vs.-rest approach and control vs. pneumonia (63.6%) in the 1-to-1 approach. Additionally, several decision trees were obtained to identify important attributes for automatic diagnosis for structured data, particularly for PTE vs. pneumonia. Based on the experiments, the structured datasets present the highest values. Results suggest using naïve Bayes and structured data to automatically diagnose PTE vs. pneumonia. Moreover, using decision trees allows the observation of some decision criteria that the medical staff could consider for diagnosis.

## 1. Introduction

### 1.1. Respiratory Diseases

Nowadays, respiratory diseases have become a point of attention in public health problems. For example, pneumonia is an infection of the lung parenchyma provoked by a bacteria or virus. This infection insidiously affects lung function by constantly reducing its operation, resulting in significant morbidity and mortality for the patients [1]. It is estimated that in the United States of America (USA), more than 1.5 million adults are hospitalized annually, with a rate of 100,000 deaths during hospitalization, from which one in three dies in less than a year [2]. In Mexico, more than 57,000 deaths associated with pneumonia were reported during the year 2020 due to the current COVID-19 pandemic [3].

According to [4], pulmonary thromboembolism (PTE) is caused by an embolus that travels and occludes the arteries of the lung. Such obstruction is the result of thrombus formation, which may result in severe or potentially lethal dyspnea [1]. PTE is the third most common cardiovascular syndrome in the world, with an estimated incidence of 0.45–0.95 per 1000 persons per year in western countries [5]. In addition, in six European countries during 2004, there were over 370,000 deaths related to Venous thromboembolism (VTE), of which 59% were PTE diagnosed after death and 7% of patients who died prematurely were correctly PTE-diagnosed before death [6]. Moreover, PTE has been the third cause of mortality in the General Hospital of the National Medical Center of the Mexican Social Security Institute (IMSS, by its acronym in Spanish) [7], with a mortality rate of 30% [8].

### 1.2. Pneumonia and PTE Diagnosis

The diagnosis of pneumonia and PTE present particular complexity considering the similarity of their symptoms, this is, cough, shortness of breath, and chest pain [9,10]; also, the given signs and symptoms are often subjective and nonspecific. For this reason, the criteria or auxiliary diagnoses specified in the guidelines [6,11] should always be complemented by the treating physician’s determination. Along with the inherent difficulty of diagnosing such diseases, medical staff must analyze, as quickly as possible, the patients’ health history, which nowadays is stored in electronic health records (EHRs). Examples of such data are laboratory tests, medical auscultation, and clinical history [12]. All this information could be helpful to medical staff to increase certainty in making a diagnosis.

This, in turn, could result in more efficient patient care at the time of hospital admission. However, to make use of EHR information, there are a least three problems. First, the amount of data increases every time a patient is admitted; second, the quality of the data is as good as the acquisition methodology; and third, the information comes from different origins, meaning that the data types are heterogeneous.

### 1.3. Computational Tools for Data Analysis

To take advantage of the EHR information, different computational methodologies could be used, for instance, Knowledge Discovery From Data (KDD) [13]. The KDD methodology is the process of generating new and useful knowledge from data sets by applying the following pipeline: cleaning, integration, data selection and transformation, data mining, pattern evaluation, and presentation of the results [14]. This pipeline guides the identification of relationship patterns between different elements in the data. Specifically, applying KDD to EHRs is intended to find patterns that, at first glance, are not evident, but that are necessary to identify factors that may be closely related to certain clinical conditions [15].

Due to the varied nature of the EHR data, one can use different strategies based on the data category, this is, structured or unstructured data. With structured data (quantitative observations), we could use traditional machine learning (ML), while for unstructured data (qualitative observations), natural language processing (NLP) is more appropriate [12,16].

### 1.4. State-of-the-Art

There is some previous work related to using computational tools for medical data analysis. For instance, the authors of [17] intended to predict diagnoses and medications categories (ICD-9) of patients by performing a multilabel classification. They use a recurrent neural network (RNN) to analyze historical data such as diagnosis codes, medication codes, or procedure codes, all extracted from EHRs belonging to 260,000 patients over eight years. They reported a recall of 79%.

A different study was presented in [18], in which the authors classified intensive care patients by analyzing clinical measurements such as body temperature, heart rate, diastolic and systolic blood pressure, and blood glucose, among others, to recognize patterns in the time series. To do so, the authors use a short-long term memory (LSTM) model. They reported an F1-score of 0.5641 and 0.4688 for classifying patients with asthma and acute respiratory distress syndrome (ARDS) (respiratory conditions), respectively.

Regarding machine-learning application in intensive care areas, the authors of [19] used both structured and unstructured data such as patient information (e.g., age and gender), vital and laboratory data (e.g., oxygen saturation and blood urea nitrogen), and clinical narrative notes (e.g., medical personal descriptions) to predict the start and finish of five invasive intervention tasks (i.e., invasive ventilation, non-invasive ventilation, vasopressors, colloid boluses, and crystalloid boluses) in the emergency department. They achieved an area under curve-receiver operating characteristic (AUC-ROC) of 0.90 using an LSTM model to predict patients with mechanical ventilation intervention.

A closer work to the one presented here is [20], where the authors propose a methodology to discriminate patients that have pneumonia from those whose do not from a sample of COVID-19-diagnosed patients. To do so, the authors used medical history and laboratory test results. They report a predictive rate of 77.1% using a backward stepwise logistic regression model and an overall predictive rate of 81.3% using a decision tree.

On the other hand, several works apply NLP methodologies to perform automatic diagnoses. For example, Liu et al. and Bagheri et al. [21,22] developed models to predict chronic diseases and cardiovascular risk prediction. The former mixed clinical text with laboratory results, while the latter used X-ray radiology reports and laboratory results. Both used a type of LSTM. The former reported a recall of 0.15 with a precision of 0.145, 0.152, and 0.025 for predicting congestive heart failure, kidney failure, and stroke. The latter obtained an F1-score above of 0.81.

Regarding respiratory conditions, the authors of [23] used NLP+SVM to diagnose patients with pneumonia, training their model with information extracted from the emergency department’s clinical notes that were tagged using ICD-9 codes. They reported a recall of 89%. On the other hand, Kaur et al. [24] identified pediatric patients that met the Asthma Predictive Index (API) criteria by analyzing EHRs with the use of NLP algorithms. The proposed model reported a sensitivity of 86% and a specificity of 98%.

For Latin American studies, Villena et al. [25] collaborated with technical and clinical experts to develop a system capable of classifying Chilean patients suffering from any of the 85 pathologies described in the national system of “Explicit Health Guarantees”. Their objective was to aid in reducing the waiting time to be attended in their corresponding health clinics. The authors used word embeddings (WE) combined with SVM, random forest, logistic regression, and multilayer perceptron. They achieved an average F1-score of 0.85 with a random forest model.

### 1.5. Aim

Considering the difficulty of making a correct diagnosis of pneumonia and PTE due to their similar symptoms, the heterogeneity and amount of data of the EHRs to be considered, and the scarcity of studies that analyze natural language text to classify these pulmonary diseases, specifically in Spanish, we propose to perform automatic classification of patients with pneumonia or pulmonary embolism through the analysis of clinical notes or laboratory results, based on the KDD procedure and the use of NLP and ML tools. This study could be useful to avoid the subjectivity of empirical clinical judgment and, by using decision tree models, provide medical experts with decision criteria that could increase their diagnosis precision.

## 2. Materials and Methods

**Figure 1:**
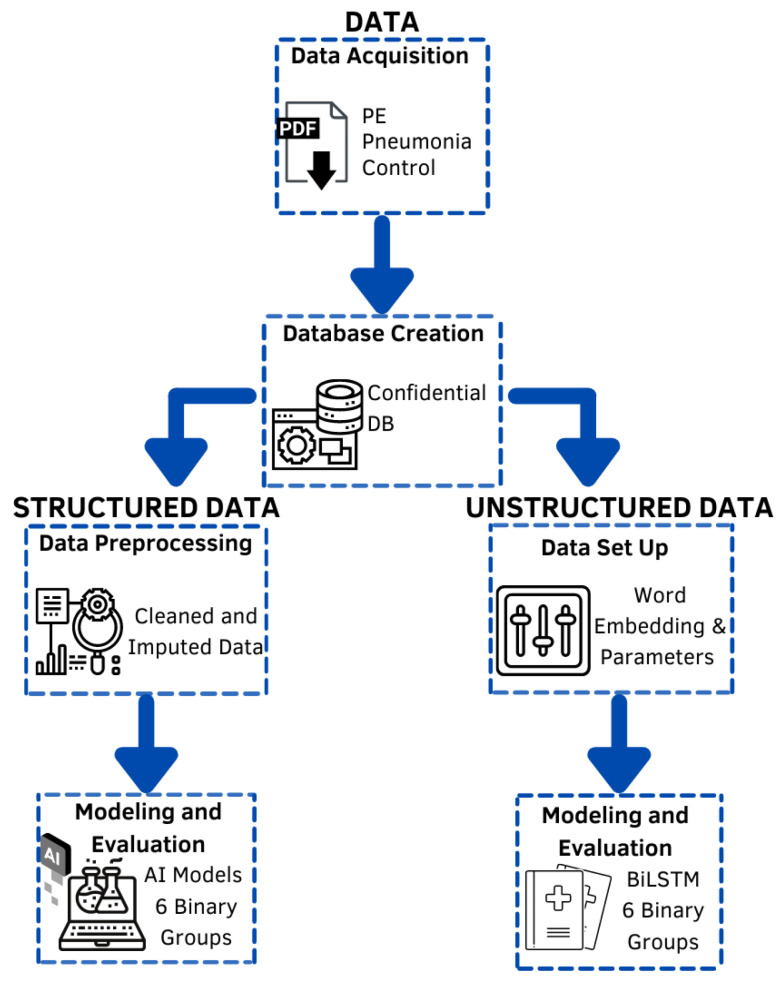
Methodological sequence.

Figure 1 depicts the methodology followed in this work. First, the data were extracted from the EHRs and stored in a relational database; then, data were split into two categories, structured and unstructured. Depending on their category, data were cleaned and prepossessed accordingly. From each data category, several machine-learning models were trained and tested using several classification evaluation methodologies.

### 2.1. Data—Data Acquisition

The EHRs were acquired following the protocol accepted on 21 April 2021 (number R-2021-785-035) by the Research and Ethics Committee of the Scientific Research National Committee of the IMSS.

According to the protocol, Table 1 shows the inclusion criteria for the collection of clinical records. All records that did not meet one or more of the previously mentioned criteria were discarded, as well as patients with diagnoses of both PTE and pneumonia. Each patient’s clinical record was defined by admission clinical notes, discharge summaries, and one or more laboratory studies requested by the emergency department.

This work corresponds to a retrospective and exploratory diagnostic study, with non-probabilistic sampling and a sample size estimated between 155 to 310 clinical records for a prevalence of 50% and a target of 80%, based on [26].

Finally, 173 clinical records were collected that met the inclusion criteria and were extracted from the EHRs in PDF format. From these, 61 had a final diagnosis of PTE, 73 of pneumonia, and 39 corresponded to control subjects.

### 2.2. Data—Database Creation

The structures of the PDF files were explored and a standard format was distinguished for each type of clinical note. It consists of two types of general structures for admission notes and discharge summaries. The structure for admission notes is shown in Table 2.

**Figure 2:**
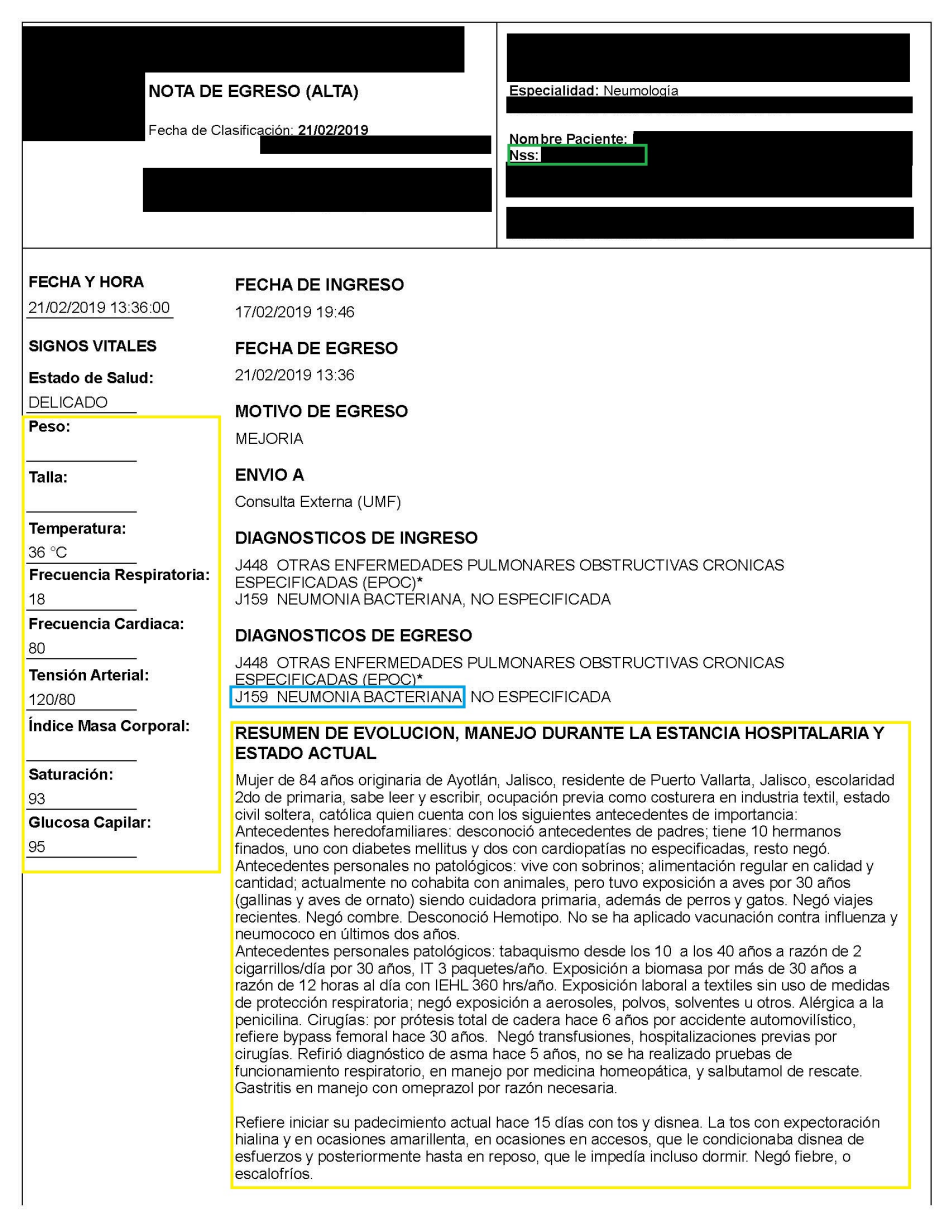
Example of discharge summary; the identity attribute used in the database is indicated in a green box. The yellow boxes highlight the structured and unstructured information used in the study and the blue box shows the diagnosis code according to ICD-10.

Table 3 shows the structure of the discharge summaries and Figure 2 shows a sample of a discharge summary where confidential information is censored and the identity attribute used in the database is indicated in a green box. The yellow boxes highlight the structured and unstructured information used in the study; in a blue box is the diagnosis code according to ICD-10.

**Figure 3:**
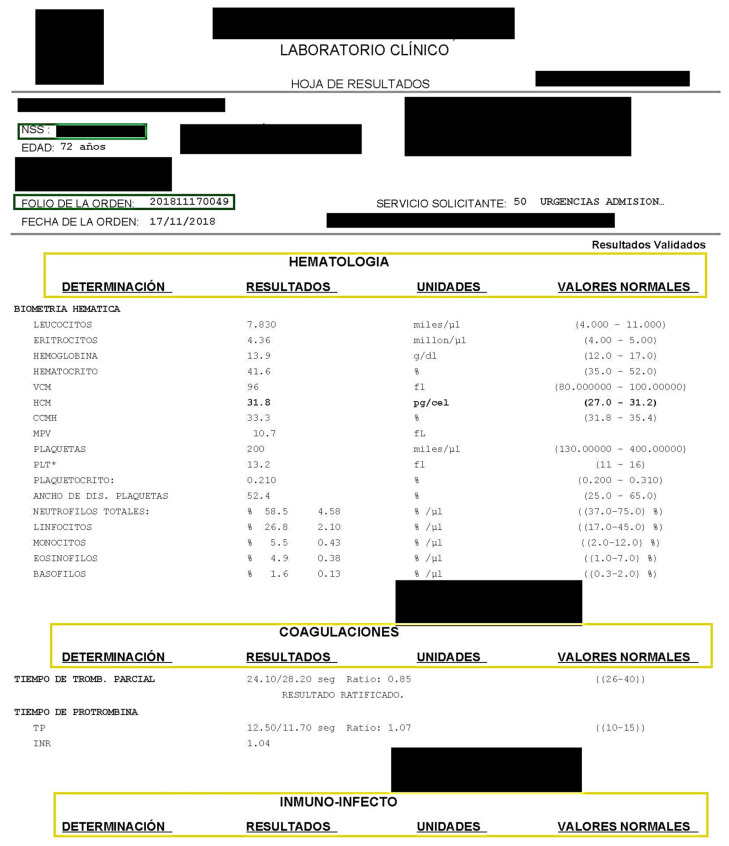
Example of laboratory study; identity attributes are highlighted in a green box and the specialized areas of the laboratory study are highlighted in a yellow box.

Table 4 and Table 5 show the laboratory studies and specialized areas corresponding to the type of laboratory study performed. Figure 3 shows an example of a laboratory study where confidential information is hidden. The identity attributes are highlighted in a green box, and the specialized areas of the laboratory study are highlighted in a blue box.

Based on the information from Table 2, Table 3, Table 4 and Table 5, a data storage structure was designed using the relational model paradigm [27] and implemented using the MySQL database manager version 8.0. To do so, a program was coded in Python version 3.8.11 with the use of the libraries pdfminer.six 20201018, pdfplumber 0.5.28, mysql-connector 2.2.9, and mysqlclient 2.0.3 to read the PDF files and extract and store the data in the database model. To identify information in the PDF files, several regular expressions were specifically designed.

The dataset obtained from the previous process is available as a Supplementary Table S1.

### 2.3. Structured Data—Data Preprocessing

First, an exploration of structured data was performed, analyzing the laboratory studies and vital signs measured during the clinical examination of the patients such as gender, weight, height, temperature, heart and respiratory rate, blood pressure, body mass index (BMI), saturation, and capillary glucose.

Using a database query, a first version of the dataset was created corresponding to the following data: vital signs (12), coagulation studies (5), hematology studies (22), immune infect studies (2), immunology studies (3), and clinical chemistry studies (47). This dataset corresponds to a matrix formed by 91 columns (attributes) and 173 row (instances). Panda library (version 1.2.4.) was used to manage the information.

Then, we looked for attributes where the number of missing values surpassed 60% concerning the total number of instances, eliminating 46 attributes. Hence, the size of the dataset decreased from 91 to 45. Table 6 presents the study and its corresponding remaining variables. The values for the instances that still contained missing values were input with the simple decision tree strategy.

A Pearson’s correlation coefficient analysis was then performed in order to identify and eliminate highly correlated variables. Pearson’s correlation reflects the linear correlation of two normal continuous variables [28], as shown in Equation (1), where *X* and *Y* contains *n* observations: $X=\{x_{0},x_{1},x_{2},\ldots ,x_{n}\}$, and $Y=\{y_{0},y_{1},y_{2},\ldots ,y_{n}\}$, with $\bar{x}$ and $\bar{y}$ corresponding to the average of *X* and *Y*, respectively. Only one of those attributes with a correlation greater than or equal to +/−0.95 was selected, under the criterion of an expert physician, as shown in Table 7.

(1) $$r=\frac{\sum_{i=1}^{n}\left(x_{i}-\bar{x}\right)\left(y_{i}-\bar{y}\right)}{\sqrt{\sum_{i=1}^{n}{\left(x_{i}-\bar{x}\right)}^{2}\sum_{i=1}^{n}{\left(y_{i}-\bar{y}\right)}^{2}}}$$

### 2.4. Structured Data—Modeling and Evaluation

To perform modeling, six machine-learning algorithms were used: decision tree (DT), random forest (RF), support vector machine (SVM), artificial neural network (ANN), naïve Bayes (NB), and AdaBoost. These models were selected because they have presented outstanding performances on several classification problems [29].

DT consists of a supervised learning method that learns from training tuples labeled by class, resulting in a flowchart-like structure, which is made up of internal nodes that denote a test on an attribute, branches that represent a test result, leaf nodes (or terminal nodes) that have a class label, and, finally, the top node that represents the most significant attribute [14]. The DT models require an attribute split criterion such as the Gini index, which considers a binary division for each attribute and measures the impurity of the data set, a data partition, or a set of training tuples (*D*), as shown in Equation (2). $p_{i}$ is the probability that a tuple in *D* belongs to class ($C_{i}$), which is estimated by $|C_{i,D}\left|/|D\right|$ [14].

(2) $$Gini\left(D\right)=1-\sum_{i=i}^{m}p_{i}^{2}$$

RF is a supervised learning method that creates a set of decision trees from a bootstrap sample with training data [29]. When developing individual trees, an arbitrary subset of attributes is drawn (hence the term “random”) from which the best attribute for the split is selected. The final model is based on the majority vote from individually developed trees in the forest [30].

SVM is an algorithm for the classification of linear and nonlinear data that uses nonlinear mapping to transform the original training data into a higher dimension and searches for the optimal linear separator hyperplane using support vectors (“essential” training tuples) and margins (defined by the support vectors) [14]. SVM requires the solution of the following optimization problem (as shown in Equation (3)).

(3) $$\begin{array}{c} \min_{\omega ,b,\xi }\\ subject\;to \end{array}\;\begin{array}{c} \frac{1}{2}w^{T}w+C\sum_{i=1}^{l}\xi_{i}\\ y_{i}\left(w^{T}\phi \left(x_{i}\right)+b\right)\geq 1-\xi_{i},\\ \xi_{i}\geq 0 \end{array}$$

Here, training vectors $x_{i}$ are mapped into a higher dimensional space by the function ϕ. SVM finds a linear separating hyperplane with the maximal margin in this higher dimensional space. C>0 is the penalty parameter of the error term. Furthermore, $K\left(x_{i},x_{j}\right)\equiv \phi {\left(x_{i}\right)}^{T}\phi \left(x_{j}\right)$ is called the kernel function [31], for example, the radial basis function (see Equation (4)), where γ is a kernel parameter.

(4) $$RBF:\;K\left(x_{i},x_{j}\right)=exp\left(-\gamma {∥x_{i}-x_{j}∥}^{2}\right),\gamma >0.$$

An ANN is a set of connected input/output units (neurons). Each input is associated with a value that weights the input. The ANN can have two or more layers. Every neuron is conformed by two operations: a weighted linear summation $w_{1}x_{1}+w_{2}x_{2}+\ldots +w_{m}x_{m}$ followed by a non-linear activation function $f\left(\cdot \right):R^{m}\to R^{o}$. The output layer receives the values from the last hidden layer and transforms them into one or several output values. The ANN is trained using the multi-layer perceptron algorithm (MLP) [14].

The MLP is a supervised learning algorithm that learns a function $f\left(\cdot \right):R^{m}\to R^{o}$ by training on a dataset, where *m* is the number of dimensions for input and *o* is the number of dimensions for output. It trains using some sort of gradient descent solver such as stochastic gradient descent (SGD) or Adam [32], updating parameters by using the gradient of the loss function with respect to each weight. These gradients are calculated using the backpropagation algorithm [33].

The NB algorithm uses all attributes to determine the probability that an instance belongs to a class, under the two assumptions that all attributes are class independent and all attributes are equally important [14]. This algorithm uses the Bayes’ theorem to calculate the probability (Pr[H|A]) of an instance belonging to a class according to Equation (5), where *A* are instances and *H* are class values.

(5) $$Pr\left[H|A\right]=\frac{Pr[A|H]Pr[H]}{Pr[A]}$$

Adaboost was formulated by Yoav Freund and Robert Schapire [34]; this method is used with other learning algorithms to improve classification performance using a classification algorithm that updates the weight of the base estimator with probability estimates, changing the distribution of the training set based on the performance of previous classifiers. An AdaBoost classifier is a meta-estimator that begins by fitting a classifier on the original dataset and then fits additional copies of the classifier on the same dataset, but where the weights of incorrectly classified instances are adjusted such that subsequent classifiers focus more on difficult cases. To do this, AdaBoost implements a new multiclass algorithm: stagewise additive modeling using a multi-class exponential loss function algorithm (SAMME) (see Equation (6)). There is also a variant of the SAMME algorithm known as SAMME.R (R for Real) that converges more quickly than SAMME and also performs slightly better than SAMME (see Equation (7)) [35].

(6) $$C\left(x\right)=\arg\max_{k}\sum_{m=1}^{M}\alpha^{\left(m\right)}\cdot I\left(T^{\left(m\right)}\left(x\right)=K\right).$$

(7) $$C\left(x\right)=\arg\max_{k}\sum_{m=1}^{M}h_{k}^{\left(m\right)}\left(x\right)$$

All classifiers’ performances were evaluated with classification accuracy (CA), F1-score, and the maximized area under the curve of the receiver operating characteristic (ROC-AUC) [14]. These evaluation metrics analyze how well a classifier can recognize positive (P) or negative (N) instances by computing metrics based on the correct predictions (TP: true positives and TF: true negatives), incorrect predictions (FP: false positives and FN: false negatives), or by a weighted average of the resulting metrics, as seen in Equation (8)–(11).

(8) $$Accuracy=\frac{TP+TN}{P+N}$$

(9) $$Precision=\frac{TP}{TP+FP}$$

(10) $$Recall=\frac{TP}{P}$$

(11) $$F1\text{-}score=\frac{2*Precision*Recall}{Precision+Recall}$$

The area under the curve indicates how well the negative and positive classes fare with respect to the decision index by measuring the entire area under the ROC curve [29].

Finally, the parameters used for each algorithm are shown in Table 8.

### 2.5. Unstructured Data—Data Set Up

To work with the clinical history of admission notes and discharge summaries, such information was obtained from the database and preprocessed using the NLTK library [36] to adapt it to the word embeddings (WE) process. WE allows the representation of words as real numerical vectors that capture semantic and syntactic relationships [37]. To do this, a preprocessing of the text was done to make them suitable for feature extraction:

1. Tokenization;
2. Stopwords removal;
3. Unnecessary characters removal;
4. Text conversion to lowercase;
5. Text stemming;
6. Text lemmatization.

The FastText library allows the generation of the WEs by representing each word as a bag of n-gram characters, thus helping to maintain the morphology of the word, enabling it to represent rare words outside the vocabulary [38].

Eight different pre-trained models made by [39] were used. Four were pre-trained with biomedical text corpora and another four with clinical text corpora; in both cases, cased and uncased CBOW and cased and uncased Skipgram architectures were used.

As a result, for each word in the clinical history, a 300-dimensional vector ranging between [−1, 1] was obtained. Then, to have just one value that represents the word, the vector’s component average was computed. It is worth noticing that the patients’ clinical histories have different lengths; thus, zero-padding should be performed for those vectors shorter than the largest vector, resulting in eight two-dimensional matrices corresponding to the vectorized clinical histories of the patients. Finally, to every matrix, an extra column vector was appended, corresponding to a given class.

Once the dataset was created, a BiLSTM model was trained to perform classification. A BiLSTM consists of two LSTM models, where one takes the input data in a forward direction and the other in a reverse direction, in order to increase the amount of information available to the network and improve the relation of a word to its context [40].

The LSTM model is used to classify and process sequential data using a set of sub-networks known as memory cells. Each memory cell preserves its state over time and regulates the flow of information through nonlinear gates. This model solves the long-term memory problem caused by the vanishing of the gradient. To do so, LSTM holds an inner condition that symbolizes the memory cell of the LSTM neuron. The inner condition state is usually augmented by recurrent gates that control the movement of the information over the cell state [41].

These gates are updated and calculated as seen in Equations (12)–(14), where $i_{t}$, $f_{t}$, and $o_{t}$ represent input, forget, and output gates, respectively [42].

(12) $$i_{t}=\tanh\left(W_{xi}x_{t}+W_{hi}h_{t-1}\right)$$

(13) $$f_{t}=\sigma \left(W_{xf}x_{t}+W_{hf}h_{t-1}\right)$$

(14) $$o_{t}=\sigma \left(W_{xo}x_{t}+W_{ho}h_{t-1}\right)$$

### 2.6. Unstructured Data—Modeling and Evaluation

The two-dimensional arrays were transformed into three-dimensional arrays to be used in a BiLSTM model. The first dimension corresponds to the samples or sequences (vectorized words), the second element is the time steps or observation points (total interrogations), and the last dimension defines the features or observations in a time step (in our case, only one feature was defined). Then, the next step was to find the best network parameters using a grid search method [43]. Table 9 shows the proposed parameters. The search was performed on a randomization dataset split 70–30% for training and testing, respectively. The process was repeated 10 times.

The BiLSTM model was trained and tested with the parameters found during the grid search according to the experiments presented in the Section 3.

## 3. Results

In the following, the results of structured and unstructured data are presented.

### 3.1. Structured Data

To perform the training and evaluation of the machine-learning algorithms, six binary datasets were created with respect to the target attribute (i.e., final diagnosis): (1) PTE vs. Pneumonia, (2) Control vs. PTE, (3) Control vs. Pneumonia, (4) PTE vs. Rest, (5) Pneumonia vs. Rest, and (6) Control vs. Diseases. Such datasets were built intending to find patterns that differentiate patients: (a) that suffer from PTE or pneumonia from those who do not (Datasets 2 and 3); (b) that suffer pneumonia from those that suffer from PTE (Dataset 1); (c) that suffer from a pulmonary disease from those who do not (Dataset 6); (d) PTE or pneumonia from control or another pulmonary disease (Datasets 4 and 5). Model evaluation was performed using area under curve (AUC-ROC), classification accuracy (CA), and F1-score. The ML models used were decision trees (DT), support vector machine (SVM), random forest (RF), artificial neural networks (ANN), naïve Bayes (NB), and AdaBoost.

Table 10 presents the results of all the ML models trained and tested with each dataset, using a five-fold cross-validation methodology. Table 11 presents the experiments evaluated using leave-one-out.

Observe that for “1-group vs. 1-group” (see Table 10), the “Pneumonia vs. Control” and the SVM algorithm obtained the best performance in both CA (83.0%) and F1-score (81.4%); additionally, the SVM obtained the second-best AUC (83.2%). Notice that, on average, this dataset presents the best CA (75.5%) and F1-score (75.5%) among all classifiers (classifier performance average per classifier, CPApC). Regarding classification algorithms, the SVM presents, on average, the highest CA (76.2%) and F1-score (74.0%) scores among all algorithms (dataset performance average per dataset, DPApC). Otherwise, the “PTE vs. pneumonia” dataset and NB obtained the highest score for AUC (85.8%) metric, the best dataset score average (AUC 80.4%), and the best classifier score average (AUC 76.0%).

On the other hand, for “1-group vs. Rest” experiments (see Table 11), considering that in this experiment the datasets are unbalanced, the AUC and F1-score are more suitable metrics to consider. Hence, the best performance was obtained for “Pneumonia vs. Rest” with NB (AUC 86.5%). The classifier performance average per dataset (CPApD) is AUC (77.7%) and the dataset performance average per classifier (DPApC) is AUC (80.1%). Notice that for the same experiment (i.e., “Pneumonia vs. Rest” with NB), the CA is around 80.1%, which could be considered high. Regarding F1-score, the best performance is achieved by AdaBoost in combination with “Control vs. Disease” dataset (F1-score 85.6%); moreover, this dataset presents the highest F1-score average among all classifiers (F1-score 79.3%). On the other hand, the F1-score for “Pneumonia vs. Rest” using NB is 80.2%, which is the highest among all classifiers and corresponds to the highest AUC (86.5%).

Additionally, a leave-one-out cross-validation was performed to consider a higher variability on each test. Table 12 and Table 13 present the results for “1-group vs. 1-group” and “1-group vs. Rest” experiments, respectively. Regarding “1-group vs. 1-group” experiments (see Table 12), note that “PTE vs. pneumonia” with NB still shows the best AUC (87.0%). In regard to CA and F-score metrics, the best scores were obtained using the “Pneumonia vs. Control” dataset with SVM, achieving 82.0% and 80.5%, respectively. On classifier performance averages, notice that the best score is still achieved by the SVM algorithm for CA (75.7%) and F1-score (73.9%). The best dataset performance average was achieved by “Pneumonia vs. Control” for CA and F1-score, with both scores being 74.7%; regarding AUC, the best score was achieved by the “PTE vs. pneumonia” dataset with 76.2%.

On the other hand, concerning “1-group vs. Rest” experiments, notice that “Control vs. Diseases” dataset along with DT algorithm reported best CA and F1-score, 88.2% and 87.5%, in that order. The best AUC, still correspond to the NB algorithm when testing “pneumonia vs. Other” dataset (86.3%). Regarding datasets performance averages per classifier, the algorithm with the higher score is DT with a CA of 77.9% and F1-score of 78.9%; on the other hand, the higher classifiers’ performance average per dataset corresponds to the dataset “Control vs. Disease” with a CA of 81.1% and F1-score: 80.0%. As to AUC, the higher datasets performance averages was achieved using the “pneumonia vs. Rest” with a 76.7%. Notice that, on the contrary to previous experimentation (Table 10, Table 11 and Table 12), in this experiment DT shows the best F1-scores to differentiate “Control vs. Diseases” and its AUC performance is 79.8% which corresponds to the highest AUC among all classifiers in “Control vs. Disease” dataset.

Based on the results obtained for the DT algorithm (see Table 13), we extended the analysis to include the decision tree graphs that achieved F1-scores closer to the average F1-scores of several trials. The objective was to provide a visual representation of the decision rules obtained from analyzing each dataset. This set of rules allows one to observe the conditions that discriminate one class from another. Table 14 depicts the average F1-score of 100 iterations (“Average F1-score” column); the rest of the columns present several evaluation metrics of the iterations with the F1-score closer to the average. Notice that the highest F1-score for the “1-group vs. 1-group” was achieved with the “PTE vs. pneumonia” dataset with a score of 0.777, obtaining a sensitivity of 0.791 and a specificity of 0.916. On the other hand, with respect to the “1-group vs. Rest”, the highest score was achieved in the “Control vs. Disease“ dataset with a score of 0.759, a sensitivity of 0.700, and a specificity of 0.400.

**Figure 4:**
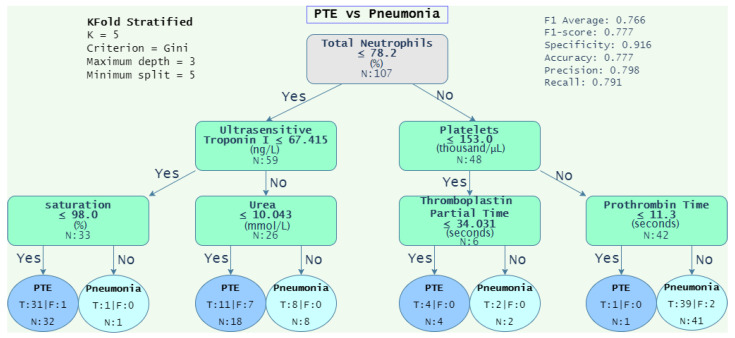
Decision tree graph of PTE vs. pneumonia, where *N* corresponds to the total of subjects analyzed, T and F are correct and incorrect classifications, respectively, and N is the total of patients evaluated.

**Figure 5:**
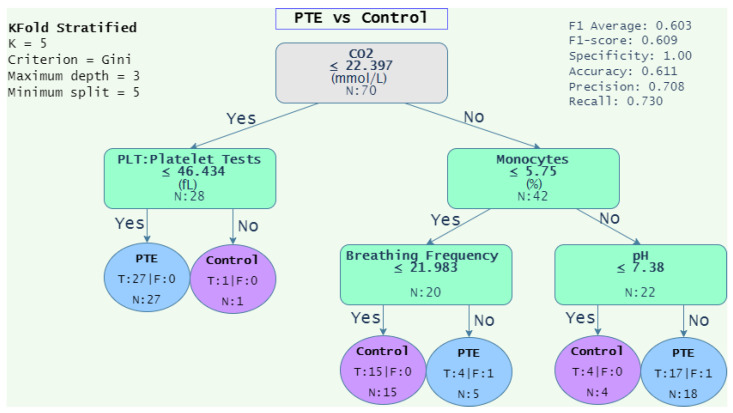
Decision tree graph of the analysis for the group of PTE versus control, where N corresponds to the total of subjects analyzed, T and F are correct and incorrect classifications, respectively, and N is the total of patients evaluated.

**Figure 6:**
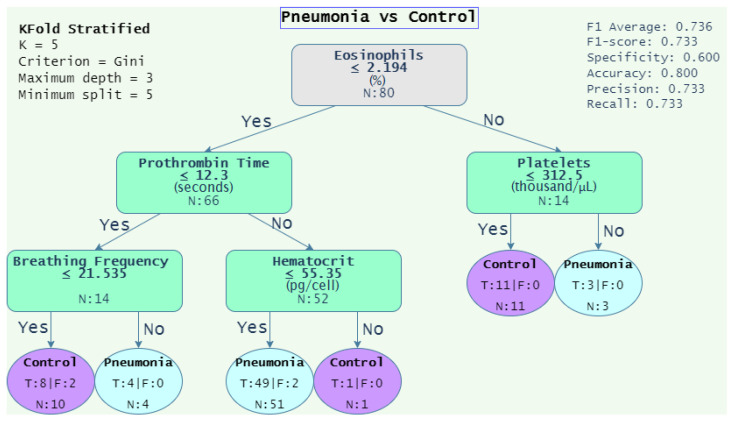
Decision tree graph of the analysis for the group of pneumonia vs. control, where N corresponds to the total of subjects analyzed, T and F are correct and incorrect classifications, respectively, and N is the total of patients evaluated.

**Figure 7:**
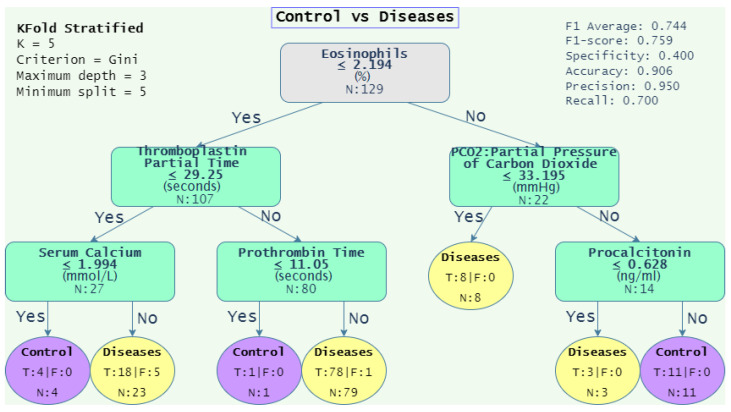
Decision tree graph of the analysis for the group of control versus diseases, where N corresponds to total subjects analyzed, T and F are correct and incorrect classifications, respectively, and N is the total patients evaluated.

Figure 4, Figure 5, Figure 6 and Figure 7 depicts the decision tree graphs corresponding to those datasets with the best F1-scores (see Table 14), that is, “PTE vs. Pneumonia”, “Control vs. Diseases”, and “Pneumonia vs. Control”, as well as “PTE vs. Control”, which does not present one of the best performances, but is of interest for specialists.

Figure 4 depicts the decision tree graph corresponding to “PTE vs. pneumonia”. It can be observed that there are tree leaves that gather 91 out of 107 patients. From that, two leaves correspond to 50 PTE patients (32 + 18) and 41 pneumonia patients. Hence, to differentiate a patient with PTE from one with pneumonia, Equation (15) is suggested by the decision tree algorithm.

(15) $$\mathrm{Condition}=\left\{\begin{array}{cc} \mathrm{PTE}, & \begin{array}{c} \;\mathrm{if}\;\mathrm{TN}\leq 78.20\;\mathrm{and}\;\\ ((\mathrm{UTI}\leq 67.41\;\mathrm{and}\;\mathrm{Sat}\leq 98)\;\mathrm{or}\;\\ \left(\mathrm{UTI}>67.41\;\mathrm{and}\;\mathrm{Ur}<10.043)\right) \end{array}\\ \mathrm{Pneumonia}, & \begin{array}{c} \mathrm{if}\;\mathrm{TN}>78.2\;\mathrm{and}\;\mathrm{Plat}>153.0\\ \;\mathrm{and}\;\mathrm{PT}>11.3 \end{array} \end{array}\right.$$

where TN = “total neutrophils”, UTI = “ultrasensitive troponin I”, “Sat = saturation”, Ur = “urea”, Plat = “platelets”, and PT = “prothrombin time”.

Additionally, Equations (16) and (17) show the decision rules extracted from Figure 5 and Figure 6, respectively. Equation (16) gathers 64 out of 70 of the patients, that is, 45 (27 + 18) for PTE and 19 (15 + 4) for control. Alternatively, Equation (17) assembles 76 out of 80 of the patients, that is, 55 (51 + 4) from pneumonia and 21 (10 + 11) from control.

(16) $$\mathrm{Condition}=\left\{\begin{array}{cc} \mathrm{PTE}, & \begin{array}{c} \mathrm{if}\;(\mathrm{CO}2\leq 22.397\;\mathrm{and}\;\mathrm{PLT}\leq 46.434)\;\mathrm{or}\;\\ (\mathrm{CO}2>22.397\;\mathrm{and}\;\mathrm{Mono}>5.75\\ \;\mathrm{and}\;\mathrm{pH}>7.38) \end{array}\\ \mathrm{Control}, & \begin{array}{c} \mathrm{if}\;\mathrm{CO}2>22.39\;\mathrm{and}\\ ((\mathrm{Mono}\leq 5.75\;\mathrm{and}\;\mathrm{BF}\leq 21.983)\;\mathrm{or}\\ (\mathrm{Mono}>5.75\;\mathrm{and}\;\mathrm{pH}\leq 7.38)) \end{array} \end{array}\right.$$

where PLT = “platelet test”, Mono = “monocytes”, BF = “breathing frequency”, pH = “potential hydrogen”, and CO2 = “carbon dioxide”.

(17) $$\mathrm{Condition}=\left\{\begin{array}{cc} \mathrm{Pneumonia}, & \begin{array}{c} \mathrm{if}\;\mathrm{Eos}\leq 2.194\;\mathrm{and}\\ ((\mathrm{ProTime}>12.3\;\mathrm{and}\;\\ \mathrm{Hema}\leq 55.35)\\ \mathrm{or}\;(\mathrm{Protime}\leq 12.3\;\mathrm{and}\;\\ \mathrm{BF}>21.535)) \end{array}\\ \mathrm{Control}, & \begin{array}{c} \mathrm{if}\;(\mathrm{Eos}\leq 2.194\;\mathrm{and}\;\\ \mathrm{ProTime}\leq 12.3\\ \mathrm{and}\;\mathrm{BF}\leq 21.535)\;\mathrm{or}\\ (\mathrm{Eos}>2.194\;\mathrm{and}\;\mathrm{Plat}\leq 312.5) \end{array} \end{array}\right.$$

where Eos = “eosinophils”, ProTime = “prothrombin time”, Hema = “hematocrit”, BF = “breathing frequency”, and Plat = “platelets”.

Finally, Figure 7 presents the DT graph for “Control vs. Disease”; the corresponding rules are shown in Equation (18).

(18) $$\mathrm{Condition}=\left\{\begin{array}{cc} \mathrm{Control}, & \begin{array}{c} \mathrm{if}\;(\mathrm{Eos}\leq 2.194\;\mathrm{and}\;\\ \mathrm{TPT}\leq 29.25\;\mathrm{and}\;\mathrm{SC}\leq 1.994)\;\mathrm{or}\;\\ (\mathrm{Ecos}>2.194\;\mathrm{and}\;\mathrm{PCO}2>33.195\\ \mathrm{and}\;\mathrm{Pro}>0.628) \end{array}\\ \mathrm{Disease}, & \begin{array}{c} \mathrm{if}\;\mathrm{Eos}\leq 2.194\;\mathrm{and}\;\\ (\mathrm{TPT}>29.25\;\mathrm{and}\;\mathrm{PT}>11.05)\;\mathrm{or}\\ (\mathrm{TPT}\leq 29.25\;\mathrm{and}\;\mathrm{SC}>1.994) \end{array} \end{array}\right.$$

where Eos = “eosinophils”, TPT = “tromboplatin partial time”, PCO2 = “partial pressure of carbon dioxide”, SC = “serum calcium”, Pro = “procalcitonin”, PT = “prothrombin time”.

### 3.2. Unstructured Data

Based on the parameters found by a grid search (see Table 9), Table 15 shows the result using the BiLSTM with six different experiments grouped by classification strategy (1-group vs. rest and 1-group vs. 1-group). The evaluation was performed using a hold-out methodology with a ratio split of 70–30%, performing 20 iterations. In every iteration, the dataset’s split was performed randomly. The best F1-score for each group of datasets is in bold.

Notice that the best performance is for differentiating control patients against patients with either pneumonia or PTE (1-group vs. Rest). The second-best is the “Control vs. Pneumonia” experiment, which is a particular case of the best-scoring experiment dataset.

## 4. Discussion

The classification algorithm performance indicates the classifiers’ capacity to differentiate between subjects with a particular condition (PTE vs. pneumonia) or between patients with a specific condition and those considered as control. Since both classes are equally important in this work, in the “1-group vs. 1-group” experiments, the AUC and CA were used as evaluation metrics. Mainly, AUC was used to find the algorithm that reports the best performance for each experiment when varying the classification threshold. On the other hand, CA, along with the validation methodology, determines the models’ average performance when varying the training and testing sets. In addition, considering that the datasets are unbalanced in the “1-group vs. Rest” cases, F1-score is taken into account. Regarding validation methodology, it will be discussed only for the leave-one-out results, since they present both higher variability in the training and testing datasets and a larger number of evaluations.

### 4.1. Structured Data

#### 4.1.1. 1-Group vs. 1-Group

Regarding the “1-group vs. 1-group” experiments (see Table 12), observe that the DPApC indicates that, on average, NB is the more adequate classification algorithm due to it corresponding to the highest AUC average over all datasets (82.4%). Particularly, the highest AUC (87.0%) was obtained by evaluating the dataset “PTE vs. pneumonia”, which indicates that there are some laboratory variables that allow for distinguishing one condition from the other. On the other hand, considering that the datasets are balanced, CA should be taken into account to observe the behavior of the classifiers while varying the training and testing datasets. Notice that contrary to the AUC experiment, the highest average CA over all datasets was achieved by the SVM (75.7%), particularly, the highest CA (82%) was achieved by the SVM classifier tested on the “Pneumonia vs. Control” dataset. In regard to the NB algorithm, the CA corresponding to “PTE vs. pneumonia” corresponds to the highest score (76.9%) among the other two datasets, “PTE vs. Control” and “Pneumonia vs. Control” with the same classification metric. Conversely, the SVM presents the second-best AUC (76.3%) corresponding to the dataset that presents the best CA, “Pneumonia vs. Control”. It is worth noticing that the worst scores in AUC and CA are presented for “PTE vs. Control”, which indicates that such classification is the more difficult to perform.

#### 4.1.2. 1-Group vs. Rest

With reference to the “1-group vs. Rest” experiments (see Table 13), notice that the highest DPApC and CPApD AUC scores corresponds the NB and “Pneumonia vs. Rest” experiments, with 80.7% and 76.7%, respectively; additionally, this combination of experiment and classification algorithm scored the higher AUC among all other options (86.3%), meaning that NB is the algorithm that obtains the best performance when varying the classification threshold. On the other hand, considering that in the “1-group vs. Rest” experiments, the datasets are unbalanced, F1-score is a good metric to observe; hence, the best DPApC and CPApD corresponds to DT and “Control vs. Diseases” experiments, with 77.9% and 80.0%, respectively, obtaining the highest score as well (87.5%).

It was also observed that the control patients present some of the best CA. obtaining 82.0% using SVM on the “Pneumonia vs. Control” dataset in the “1-group vs. 1-group” experiment (see Table 12), and an F1-score of 87.5% using DT on the “Control vs. Disease” dataset in the “1-group vs. Rest” experiments (see Table 13). It is noticeable that in all the experiments that involved “Pneumonia vs. Control”, using either five-fold cross-validation or leave-one-out, the same classification algorithms obtained better performances using this dataset than training with the “PTE vs. Control” dataset. From this, it is feasible to deduce that the laboratory variables used in this work to describe the PTE condition are not enough, which agrees with the guidelines listed in [6] that advise having a compatible clinical picture such as shortness of breath, chest pain, cough, hemoptysis, and tachypnea. In addition, arterial blood gas and cardiac enzyme studies are required to determine the severity of the disease and a specialized imaging study of pulmonary angiotomography is needed to confirm the diagnosis. This latter study is the gold standard for the diagnosis of PTE, since it evidences the thrombus in the pulmonary arteries. On the other hand, the diagnosis of pneumonia is advised to be carried out through shortness of breath, fever, chills, and cough; a simpler imaging study, that is, a chest X-ray to corroborate the diagnosis; and laboratory studies of hematology and clinical chemistry, as indicated by [11]. Therefore, the complexity of the pulmonary thromboembolism diagnosis compared with pneumonia is evident.

To the best of our knowledge, there is no previous work regarding the classification of PTE vs. pneumonia; the closest work found was [20], where the author intended to discriminate pneumonia vs. non-pneumonia in patients with COVID-19. The authors of [20] reported a predictive rate of 77.1% using a backward stepwise logistic regression model, which is lower than the CA of 82.0% scored by the SVM and near to the CA of 76.9% scored by the NB both using the “Pneumonia vs. Control” (see Table 12). Additionally, the authors of [20] reported the use of the decision tree algorithm on their classification task; nonetheless, it is not clear which parameters were used for training the model, nor what validation methodology was used. This is contrary to the present work, where all decision tree parameters are presented, as well as the decision rules and validation methodology. Additionally, their sample (50 patients) was lower than that of the present work (173 patients).

#### 4.1.3. Decision Rules

Regarding the decision rules obtained from the decision trees, Figure 4 shows the result of the DT model trained with “PTE vs. Pneumonia” dataset. It is observed that neutrophils above 78.2 are associated with infectious processes such as pneumonia, which is derived from the activation of inflammatory cells during an inflammatory process. This is why platelets are above 153 and why patients with PTE present lower total neutrophils [44]. Furthermore, ultrasensitive troponin I is used as a criterion for the stratification of the severity of PTE, so it is expected that patients are in both deviations of the graph, as they show low or high values based on the severity of the disease [6]. When the infectious process is severe, it may present with elevated prothrombin time (>11.3), as observed in the 41 patients classified with pneumonia by the model [45]. Finally, those patients with lower neutrophils (<78.2), could be elderly patients, since they may not have elevated neutrophils or elevated troponin and urea, which indicate a more severe disease, since these patients may have a diffusion of several organs such as the heart and kidneys [46]. However, we can appreciate an imbalance in the classification of subjects with PTE by urea, where 7 subjects out of 18 are misclassified. This is because urea is an attribute that is used to classify the severity of pneumonia according to the CURB-65 scale [47], so the subjects misclassified with PTE are actually subjects with non-severe pneumonia.

On the other hand, it was observed in Figure 5, “PTE vs. Control”, that the decision begins with the value of CO2 ≤ 22,397 mmHg. It is known that pulmonary embolism causes an increase in dead space due to a ventilation–perfusion imbalance, causing an increase in arterial CO2 and activating medullary chemoreceptors that increase minute ventilation, decreasing arterial CO2 and causing respiratory alkalosis, which is frequently observed in patients with pulmonary thromboembolism [48]. The platelet count for patients with PTE has values lower than 46.4. However, a value near 15 is still normal for healthy patients [49].

In Figure 6 for “Pneumonia vs. Control”, the decision begins with the percentage of eosinophils, which is associated with increased risk of pneumonia in patients with chronic obstructive pulmonary disease [50]. A higher respiratory rate inclines the decision to pneumonia; the respiratory rate is even a marker of severity included in some scales such as CURB-65 [47]. It was observed that most of the patients with pneumonia have hematocrit values below 55.35; this is because the control subjects are patients with various degrees of obesity whose disease is usually associated with insulin resistance that increases the level of hemoglobin and hematocrit [51]. Obesity is also associated with polyglobulia, which is a disease with elevated hematocrit [52].

The DT model for “Control vs. Diseases” (see Figure 7) shows that low eosinophils (<2.1) are present in acute respiratory pathologies, as in patients with severe COVID-19, who have presented values of up to zero eosinophils [53].

### 4.2. Unstructured Data

In the case of unstructured data analysis, there are no other works, presumably, that address the classification problem of PTE vs. pneumonia using BiLSTM, so a direct comparison is not available. However, some works use natural language processing applied to different conditions; for example, the authors of [21] reported a BiLSTM that achieved a recall of 0.15 with a precision of 0.145, 0.152, and 0.025 for predicting congestive heart failure, kidney failure, and stroke. Compared with the present work, our proposal overcomes their results by achieving 0.657 and 0.653 for precision and recall, respectively, to predict “pneumonia vs. Control”; and 0.567 for precision and recall predicting “PTE vs. pneumonia”. On the contrary, the authors of [22] reported an F1-score of around 0.84 when doing cardiovascular risk prediction; such a result is greater than the highest F1-score presented in this work, which was achieved when training with the dataset “Control vs. Diseases”. This behavior is common, since it is known that the same classification methodologies do not always work for every problem. In addition, other variables that could affect the performance of our model are the number of clinical notes analyzed, as well as the limited availability of training corpus for the analysis of clinical text in Spanish [54]; even when using corpus in Spanish, if the corpus is from a different region, for example, Spain, such differences will impose certain language limitations, which will be reflected in the BiLSTM model. This is the case in the present work.

## 5. Conclusions

For medical staff, pneumonia and pulmonary thromboembolism diagnosis is a challenge due to the similarity of symptoms. The information contained in electronic health records is helpful to carry out that diagnosis; however, this information is extensive and heterogeneous, making it complicated for an expert to analyze it all. In this work, we performed automatic classification of these respiratory diseases using machine-learning techniques and data obtained from the EHRs, considering structured (laboratory information) and unstructured data (patient clinical history in Spanish).

Regarding the structured data or laboratory variables, the obtained results in the 1-vs.-1 experiment showed that using the naïve Bayes model, it is possible to distinguish PTE vs. pneumonia with more precision, according to the AUC metric. In the 1-vs.-Rest approach, it was observed that there is greater complexity in diagnosing PTE than pneumonia. On the other hand, even though the decision tree algorithm does not present the best performance, it has the advantage of a visual description that might be used by the specialist to perform a diagnosis; in this sense, the model considered the neutrophils variable as the most important feature to distinguish between PTE and pneumonia.

Concerning unstructured data or clinical text, the classification of PTE vs. pneumonia using the BiLSTM model does not present good performance, achieving a precision of 57.6%. This low performance could be related to the limited availability of training corpus for the analysis of clinical text in Spanish.

Some limitations can be found in this work, for example, the use of WE built using Spanish from Spain. It would be preferable to build a WE from scratch using notes that use Mexican Spanish. Furthermore, the number of records expected was between 155 to 310 per condition; however, it was not possible to obtain the desired number of patients per condition, so a greater amount of EHRs will be used in future works. Only the “1-group vs. 1-group” and “1-group vs. Rest” strategies were explored; building a three-class model would be interesting. Finally, a combination of structured data and unstructured data would be interesting for future work.

## Figures and Tables

**Table 1:** Inclusion criteria for the collection of clinical records.

Subjects with PTE or Pneumonia	Control Subjects
Patients over 18 years old	Patients over 18 years old
Patients with an admission note from the emergency department.	Patients without a final diagnosis of pneumonia or pulmonary embolism
Patients with one or more laboratory studies requested by the emergency department	Admission notes for preoperative assessment for bariatric surgery
Laboratory studies not older than one week with respect to the patient’s admission note	Patients with one or more laboratory studies for pre-surgical assessment of bariatric surgery
Laboratory studies with one or more studies of blood biometry, procalcitonin, blood chemistry, serum electrolytes, coagulation times, and/or arterial blood gases	Laboratory studies with one or more studies of blood biometry, procalcitonin, blood chemistry, serum electrolytes, coagulation times, and/or arterial blood gases
Patients with discharge summary from pulmonology	Discharge summary for pre-surgical assessment for bariatric surgery
Discharges summaries with final diagnosis of PTE or pneumonia	Discharge summary with final diagnosis of obesity due to excess calories
Final diagnosis according to ICD-10 classification	Final diagnosis according to ICD-10 classification
Clinical records from the year 2017 to 2022	Clinical records from the year 2017 to 2022

**Table 2:** Fields extracted from the entry notes. Ql: qualitative, Qt: quantitative, N: nominal, T: text, D: discrete, C: continuous.

Field	Nature	Type	Field	Nature	Type
NSS (unique identifier)	Ql	N	Health indications and status	Ql	T
Date of admission	Qt	D	Weight	Qt	C
Subject’s gender	Ql	D	Height	Qt	C
Admission specialty	Ql	T	Temperature	Qt	C
Reason for admission	Ql	T	Respiratory rate	Qt	D
Interrogation	Ql	T	Blood pressure	Qt	D
Initial diagnosis	Ql	T	BMI (body mass index)	Qt	C
Treatment plan	Ql	T	Peripheral oxygen saturation	Qt	D
Prognosis	Ql	T	Capillary glucose	Qt	D

**Table 3:** Fields extracted from discharges summaries. Ql: qualitative, Qt: quantitative, N: nominal, T: text, D: discrete, C: continuous.

Field	Nature	Type	Field	Nature	Type
NSS (unique identifier)	Ql	N	Health indications and status	Ql	T
Date of admission	Qt	D	Prognosis of health	Ql	T
Date of discharge	Qt	D	Health status	Ql	T
Subject’s gender	Ql	T	Diagnosis discharge/demise	Ql	T
Specialty of discharge	Ql	T	Weight	Qt	C
Reason for egress	Ql	T	Size	Qt	C
Referral to specialty	Ql	T	Temperature	Qt	C
Admission diagnosis	Ql	T	Respiratory rate	Qt	D
Summary of progress	Ql	T	Blood pressure	Qt	D
Treatment plan	Ql	T	BMI (body mass index)	Qt	C
Recommendations	Ql	T	Peripheral oxygen saturation	Qt	D
Risk factors	Ql	T	Capillary blood glucose	Qt	D

**Table 4:** Fields extracted from laboratory studies. Ql: qualitative, qt: quantitative, N: nominal, T: text, D: discrete.

Field	Nature	Type	Extracted Field	Nature	Type
NSS (unique identifier)	Ql	N	Patient’s age	Qt	D
Order folio requested	Ql	N	Qualitative service	Ql	T
Date of order	Qt	D			

**Table 5:** Specialized fields extracted from laboratory studies. Ql: qualitative, Qt: quantitative, D: discrete, C: continuous, T: text.

Field	Operational Definition	Nature	Type
Determination	Contains the name of the variable	Ql	T
Result	Contains the value of the variable	Qt	D/C
Unit	Contains the unit of the variable	Ql	T
Normal value	Contains the limiting values of the variable	Qt	D/C

**Table 6:** Variables resulting from the elimination of missing data.

Variables	Studies	Variables	Studies
Dimer II	Coagulation	Plateletocrit	Hematology
Thromboplastin partial time	Coagulation	Platelet Count (PLT)	Hematology
Prothrombin time	Coagulation	Red cell blood distribution width (RDW)	Hematology
Age	Vital signs	Mean corpuscular volume (MCV)	Hematology
Breathing frequency	Vital signs	Procalcitonin	Immune infect
Gender	Vital signs	High-sensitive troponin I	Immune infect
Diastolic blood pressure	Vital signs	Serum calcium	Clinical chemistry
Systolic blood pressure	Vital signs	Chlorine	Clinical chemistry
Saturation	Vital signs	CO2	Clinical chemistry
Temperature	Vital signs	Serum creatinine	Clinical chemistry
Platelet distribution width (PDW)	Hematology	Base excess	Clinical chemistry
Basophils	Hematology	Phosphorus	Clinical chemistry
Mean corpuscular hemoglobin concentration (MCHC)	Hematology	Blood glucose	Clinical chemistry
Eosinophils	Hematology	HCO3	Clinical chemistry
Erythrocytes	Hematology	Magnesium	Clinical chemistry
Mean corpuscular hemoglobin (MCH)	Hematology	PCO2	Clinical chemistry
Hematocrit	Hematology	pH	Clinical chemistry
Leukocytes	Hematology	PO2	Clinical chemistry
Lymphocytes	Hematology	potassium	Clinical chemistry
Monocytes	Hematology	O2 saturation	Clinical chemistry
Mean platelet volume (MPV)	Hematology	Sodium	Clinical chemistry
Total neutrophils	Hematology	Urea	Clinical chemistry
Platelets	Hematology		

**Table 7:** Attributes with Pearson correlation greater than +/−0.95.

r Value	Selected	Discarded
+1	Urea	Calculated Urea
+0.965	Hematocrit	Hemoglobin
+0.965	PT: Prothrombin Time	INR: International Normalized Ratio

**Table 8:** Training step parameters by algorithm.

Algorithm	Parameter	Value
Decision tree	Minimum number of instances in leaves	3
	Limit of subsets splits	5
	Maximal tree depth	3
	Majority reaches (%)	95
Random forest	Number of trees	5
	Limit of subsets splits	5
Support vector machine	Cost	1
	Regression loss epsilon	0.10
	Kernel	RBF
	Numerical tolerance	0.001
	Iteration limit	100
Neural networks	Neurons in hidden layers	10, 6
	Activation	Tanh
	Solver	Adam
	Regularization	0.03
	Maximal iterations	2500
Adaboost	Base of estimator	Tree
	Number of estimators	50
	Learning rate	1
	Classification algorithm	SAMME.R
	Regression loss function	Linear

**Table 9:** Proposed parameters for BiLSTM.

Parameter	Proposed Values	Selected Values
Optimizer	[‘adam’, ‘SGD’]	SGD
Learning rate	[0.01, 0.025, 0.05, 0.1, 0.5]	0.1
Momentum	[0.01, 0.025, 0.05, 0.075, 0.1, 0.5]	0.1
Neurons	[5, 10, 20, 50, 100]	50
Density	[1, 2, 3, 4, 5]	1
Epochs	[5, 10, 25, 50, 100]	25

**Table 10:** The “1-group vs. 1-group” experiments evaluated using a five-fold cross-validation methodology. Values are presented in percentages. The first element of the dataset is the positive attribute.

Metric	Dataset	DT	SVM	RF	ANN	NB	AdaBoost	CPApD ^1^
AUC	PTE vs. Control	59.1	63.1	70.1	54.8	71.8	77.2	66
	Pneumonia vs. Control	61.1	77.55	78.5	78.2	83.7	71.9	75.2
	PTE vs. Pneumonia	69.0	83.2	71.8	81.5	85.8 *	64.6	76.0
	DPApC ^2^	63.1	74.6	73.5	71.5	80.4	71.2	
CA	PTE vs. Control	61.4	71.6	71.6	61.4	61.4	78.4	67.6
	Pneumonia vs. Control	65.0	83.0 *	79.0	77.0	73.0	76.0	75.5
	PTE vs. Pneumonia	66.4	73.9	66.4	70.9	79.9	64.2	70.3
	DPApC ^2^	64.3	76.2	72.3	69.8	71.4	72.9	
F1-score	PTE vs. Control	62.5	66.8	67.7	61.4	62.8	78.9	66.7
	Pneumonia vs. Control	65.6	81.4 *	77.9	76.9	74.5	76.5	75.5
	PTE vs. Pneumonia	66.5	73.8	66.3	70.9	79.9	64.2	70.3
	DPApC ^2^	64.9	74.0	70.6	69.7	72.4	73.2	

* Best performance per metric. ^1^ Classifier performance average per dataset. ^2^ Dataset performance average per classifier.

**Table 11:** The “1-group vs. Rest” experiments evaluated using a five-fold cross-validation methodology. Values are presented in percentages. The first element of the dataset is the positive attribute.

Metric	Dataset	DT	SVM	RF	ANN	NB	AdaBoost	CPApD ^1^
AUC	PTE vs. Rest	71.9	75.9	75.2	75.4	79.4	61.9	73.3
	Pneumonia vs. Rest	76.1	81.8	77.9	79.5	86.5 *	64.1	77.7
	Control vs. Diseases	69.5	69.8	74.3	67.6	74.3	71.1	71.1
	DPApC ^2^	72.5	75.8	75.8	74.2	80.1	65.7	
CA	PTE vs. Rest	71.4	69.6	72.0	69.6	69.6	64.6	69.5
	Pneumonia vs. Rest	68.9	71.4	72.7	73.3	80.1	64.6	71.8
	Control vs. Diseases	82.6	82.6	85.1	80.1	65.8	86.3 *	80.4
	DPApC ^2^	74.3	74.5	76.6	74.3	71.8	71.8	
F1-score	PTE vs. Rest	70.1	66.1	71.2	70.0	70.0	64.4	68.6
	Pneumonia vs. Rest	68.5	71.3	72.5	73.3	80.2	64.5	71.7
	Control vs. Diseases	81.3	75.3	83.3	80.1	70.1	85.6 *	79.3
	DPApC ^2^	73.3	70.9	75.7	74.5	73.4	71.5	

* Best performance per metric. ^1^ Classifiers’ performance average per dataset. ^2^ Dataset performance average per classifier.

**Table 12:** The “1-group vs. 1-group” experiments evaluated using a leave-one-out methodology. Values are presented in percentages. The first element of the dataset is the positive attribute.

Metric	Datasets	DT	SVM	RF	ANN	NB	AdaBoost	CPApD ^1^
AUC	PTE vs. Control	55.0	66.8	59.5	58.3	75.1	67.8	63.8
	Pneumonia vs. Control	57.3	76.3	75.0	74.4	85.2	65.7	72.3
	PTE vs. Pneumonia	69.1	80.1	73.4	79.0	87.0 *	68.8	76.2
	DPApC ^2^	60.5	74.4	69.3	70.6	82.4	67.4	
CA	PTE vs. Control	62.5	70.5	64.8	67.0	67.0	68.2	66.7
	Pneumonia vs. Control	69.0	82.0 *	80.0	75.0	75.0	67.0	74.7
	PTE vs. Pneumonia	60.4	74.6	65.7	72.4	76.9	69.4	69.9
	DPApC ^2^	64.0	75.7	70.2	71.5	73.0	68.2	
F1-score	PTE vs. Control	58.4	66.8	62.5	66.5	68.3	69.2	65.3
	Pneumonia vs. Control	69.5	80.5	78.4	75.1	76.3	68.6	74.7
	PTE vs. Pneumonia	60.5	74.5	65.7	72.4	76.9	69.3	69.9
	DPApC ^2^	62.8	73.9	68.9	71.3	73.8	69.0	

* Best performance per metric. ^1^ Classifiers’ performance average per dataset. ^2^ Dataset performance average per classifier.

**Table 13:** The “1-group vs. Rest” experiments evaluated using a leave-one-out methodology. Values are presented in percentages. The first element of the dataset is the positive attribute.

Metric	Datasets	DT	SVM	RF	ANN	NB	AdaBoost	CPApD ^1^
AUC	PTE vs. Rest	74.0	74.8	76.0	75.2	79.7	67.8	74.6
	Pneumonia vs. Rest	73.7	81.9	73.1	79.1	86.3 *	66.3	76.7
	Control vs. Diseases	79.8	67.0	68.6	64.2	76.0	73.7	71.6
	DPApC ^2^	75.8	74.6	72.6	72.8	80.7	69.3	
CA	PTE vs. Rest	75.2	69.6	69.6	70.8	74.5	69.6	71.6
	Pneumonia vs. Rest	73.3	72.7	65.8	69.6	80.1	66.5	71.3
	Control vs. Diseases	88.2	83.2	83.9	77.0	68.3	85.7	81.1
	DPApC ^2^	78.9	75.2	73.1	72.5	74.3	73.9	
F1-score	PTE vs. Rest	73.7	66.1	69.0	70.9	74.9	69.6	70.7
	Pneumonia vs. Rest	72.5	72.6	65.7	69.6	80.2	66.5	71.2
	Control vs. Diseases	87.5 *	75.6	81.4	77.8	72.2	85.6	80.0
	DPApC ^2^	77.9	71.4	72.0	72.8	75.8	73.9	

* Best performance per metric. ^1^ Classifiers’ performance average per dataset. ^2^ Dataset performance average per classifier.

**Table 14:** Average decision tree models for all datasets using five-fold stratified cross-validation. Spec = specificity, CA = accuracy, Pr = precision, Sens = sensitivity. The first element of the dataset is the positive attribute.

Dataset	Average F1-Score	F1-Score	Spec	CA	Pr	Sens
PTE vs. Control	0.603	0.609	1.000	0.611	0.708	0.730
Pneumonia vs. Control	0.736	0.733	0.600	0.800	0.733	0.733
PTE vs. Pneumonia	0.766	0.777	0.916	0.777	0.798	0.791
PTE vs. Rest	0.657	0.727	0.538	0.757	0.763	0.719
Pneumonia vs. Rest	0.636	0.619	0.571	0.625	0.619	0.619
Control vs. Diseases	0.744	0.759	0.400	0.906	0.950	0.700

**Table 15:** Average results (percentage) of the BiLSTM model with unstructured data for six different binary datasets.

Group	Accuracy	Precision	Recall	F1-Score	AUC
Control vs. Diseases	61.3	77.1	71.6	**72.7**	50.3
PTE vs. Rest	51.3	60.4	63.2	60.5	46.3
Pneumonia vs. Rest	48.6	56.3	60.5	57.0	46.6
Control vs. PTE	52.9	64.0	57.0	58.5	52.1
Control vs. Pneumonia	54.3	65.7	65.3	**63.6**	48.4
PTE vs. Pneumonia	51.7	56.7	56.7	55.9	47.9

## Data Availability

The data presented in this study are available as supplementary material.

## Supplementary Materials

The following supporting information can be downloaded at: https://www.mdpi.com/article/10.3390/diagnostics12102536/s1, Table S1: Patients dataset.

## Abbreviations

The following abbreviations are used in this manuscript:

USA	United States of America
PTE	Pulmonary Thromboembolism
VTE	Venous Thromboembolism
IMSS	Mexican Social Security Institute (by its acronym in Spanish)
EHR	Electronic Health Records
KDD	Knowledge Discovery From Data
ML	Machine Learning
NLP	Natural Language Processing
ICD-9	International Classification of Disease, Ninth Revision
ICD-10	International Classification of Disease, Tenth Revision
RNN	Recurrent Neural Network
LSTM	Long-Short Term Memory
ARDS	Respiratory Distress Syndrome
AUC-ROC	Area Under Curve-Receiver Operating Characteristic
SVM	Support Vector Machine
API	Asthma Predictive Index
WE	Word Embeddings
RF	Random Forest
PDF	Portable Document Format
NB	Naïve Bayes
NSS	Social Security Number (by its acronym in Spanish)
DT	Decision Tree
ANN	Artificial Neural Network
SGD	Stochastic Gradient Descent
CBOW	Continuous Bag of Words
BiLSTM	Bidirectional Long-Short Term Memory
CA	Classification Accuracy
DPApC	Dataset Performance Average per Classifier
CPApD	Classifier Performance Average per Dataset
TN	Total Neutrophils
UTI	Ultrasensitive Troponin I
Sat	Saturation
Ur	Urea
Plat	Platelets
PT	Prothrombin Time
PLT	Platelet Test
Mono	Monocytes
BF	Breathing Frequency
pH	Potential Hydrogen
CO2	Carbon Dioxide
Eos	Eosinophils
ProTime	Prothrombin Time
Hema	Hematocrit
TPT	Tromboplatin Partial Time
PCO2	Partial Pressure of Carbon Dioxide
SC	Serum Calcium
Pro	Procalcitonin
